# Facing Mortality: Spiritual Needs, Psychological Distress, and Death Anxiety in Hemodialysis Patients in Eastern Turkey

**DOI:** 10.1007/s10943-026-02716-0

**Published:** 2026-06-27

**Authors:** Ebubekir Kaplan, Cemile Hurrem Ayhan, Mehmet Cihad Aktas, Huseyin Ataş, Fuat Tanhan

**Affiliations:** 1 Department of Fundamental of Nursing, Faculty of Health Science, Van Yuzuncu Yil University, Van, Turkey; 2 Department of Psychiatric Nursing, Faculty of Health Science, Van Yuzuncu Yil University, Campus Tusba, 65030 Van, Turkey; 3 Department of Psychiatric Nursing, Faculty of Health Science, Van Yuzuncu Yil University, Van, Turkey; 4 Department of Psychiatric and Mental Health Nursing, Institute of Health Science, Van Yuzuncu Yil University, Van, Turkey; 5 Department of Educational Science, Head of the Department of Guidance and Psychological Counseling, Faculty of Education, Van Yuzuncu Yil University, Van, Turkey

**Keywords:** Spiritual needs, Depression, Stress, Anxiety, Death anxiety

## Abstract

This descriptive and correlational study was conducted with 131 patients undergoing hemodialysis in Eastern Turkey to examine levels of spiritual needs, psychological distress, and death anxiety, as well as the relationships among these variables and predictors of death anxiety. The participants reported mild levels of depression and stress and moderate levels of anxiety. The mean score on the Templer Death Anxiety Scale indicated a high level of death anxiety (M = 8.80, SD = 1.88). Spiritual needs, assessed via a contamination-adjusted measure, were positively associated with death anxiety but were not significantly related to depression, anxiety, or stress. Hierarchical regression analysis revealed that spiritual needs significantly predict death anxiety even after controlling for psychological distress. These findings suggest that spiritual needs represent a distinct existential dimension in hemodialysis patients and play a unique role in shaping fear of death. Integrating spiritually sensitive and existentially informed care into hemodialysis services may help reduce death-related distress and support psychological well-being in this population.

## Introduction

Chronic renal failure is a progressive and irreversible condition that profoundly affects physical functioning, psychological well-being, and overall quality of life. Globally, approximately 8–10% of the adult population is estimated to exhibit some degree of renal impairment, and millions of individuals die prematurely each year because of complications related to chronic kidney disease (CKD) (Brücke et al., 2016; Lv & Zhan, 2019). As CKD progresses, many patients progress to end-stage renal disease (ESRD), a stage at which renal replacement therapy-either dialysis or kidney transplantation-becomes essential for survival. Among the available treatment modalities, hemodialysis (HD) remains the most widely used approach in many countries (Shi et al., 2022). In Turkey, more than 70,000 individuals currently receive dialysis treatment, highlighting the growing public health burden of kidney failure.

Although hemodialysis plays a critical life-sustaining role by removing metabolic waste products and excess fluid from the bloodstream, it also imposes substantial physical, emotional, and social demands. Patients must adapt to strict treatment schedules, dietary and fluid restrictions, reduced physical capacity, and ongoing dependence on medical technology. These challenges often disrupt daily routines, employment, family roles, and social relationships, contributing to feelings of loss of independence, uncertainty about the future, and diminished life satisfaction (Gerogianni et al., 2018). Over time, the cumulative burden of living with a chronic, life-threatening condition and undergoing repetitive invasive procedures may erode psychological resilience and increase vulnerability to mental health problems.

Psychological distress, including symptoms of depression, anxiety, and stress, is highly prevalent among individuals undergoing hemodialysis and represents a major concern for both patients and healthcare providers.

According to the World Health Organization (2023a, 2023b), depression affects approximately 3.8% of the global population, whereas anxiety disorders affect nearly 4.0% of individuals worldwide. However, patients receiving dialysis experience substantially higher rates of depression and anxiety than the general population does. A large meta-analysis involving more than 55,000 participants reported prevalence rates of depressive symptoms reaching 39.3% among dialysis patients when assessed by rating scales (Palmer et al., 2013). Psychological distress in this population has been associated with poorer adherence to treatment regimens, reduced quality of life, increased hospitalization, and adverse health outcomes (Shafi & Shafi, 2017). Older adults receiving hemodialysis may be particularly vulnerable to age-related physical decline, comorbid conditions, social isolation, and the cumulative strain of long-term treatment (Khan et al., 2022).

In addition to experiencing general psychological distress, hemodialysis patients frequently face profound existential concerns related to illness, uncertainty, and mortality. One of the most significant emotional burdens in this population is death anxiety, which is defined as persistent fear, worry, or preoccupation with death and dying. Patients who are living with ESRD are required to be continuously aware of their life-threatening condition, depend on life-sustaining technology, and be uncertain about disease progression. The repetitive and invasive nature of hemodialysis, coupled with the possibility of sudden health deterioration, may intensify fears related to suffering, loss of control, and the prospect of death. Empirical studies indicate that a substantial proportion of hemodialysis patients experience moderate to severe levels of death anxiety. For example, Dewina et al. (2018) reported that approximately 47.9% of hemodialysis patients exhibited moderate levels of death anxiety. Furthermore, external stressors, such as the COVID-19 pandemic, have been shown to exacerbate death-related fears and broader psychological distress in this vulnerable population (Ibrahim et al., 2023). Elevated death anxiety has also been linked to increased stress, anxiety, and depressive symptoms and poorer quality of life (Ibrahim et al., 2023).

In addition to psychological and existential challenges, spiritual concerns may play an important role in how individuals cope with chronic illness and the prospect of mortality. Facing prolonged illness often prompts reflection on meaning, purpose, hope, faith, and transcendence. In this context, spiritual needs, such as the desire for meaning in life, inner peace, comfort through religious or spiritual beliefs, and connection with transcendent values, may become increasingly salient. Patients undergoing hemodialysis frequently report spiritual struggles and unmet spiritual needs that influence their emotional well-being, coping processes, and perceptions of illness (Siwi et al., 2020). Research in chronic illness populations suggests that spiritual and meaning-oriented resources may buffer against psychological distress and existential anxiety (Ghiasi et al., 2021; Shirkavand et al., 2018). However, empirical evidence specifically examining the contribution of spiritual needs, which are distinct from general spirituality or spiritual well-being, to death anxiety among hemodialysis patients remains limited.

Cultural and regional contexts may further shape how individuals experience illness, spirituality, and death-related fears. In Eastern Turkey, where religious beliefs, family structures, and cultural attitudes toward illness and mortality may differ from those in Western countries, spiritual needs and existential concerns could play a particularly significant role in the psychological adjustment to chronic disease. However, research exploring the interrelationships among spiritual needs, psychological distress, and death anxiety in hemodialysis patients in this region remains scarce.

Recent literature published over the past five years has increasingly highlighted the psychological and existential burdens associated with hemodialysis, including heightened risks of depression, anxiety, and death-related fears (Ibrahim et al., 2023; Khan et al., 2022). Parallel research on chronic illness and psycho-oncology has underscored the clinical relevance of spiritual needs and meaning-making processes in shaping mental health outcomes (Ghiasi et al., 2021; Siwi et al., 2020). Nevertheless, further research is needed to better understand whether spiritual needs uniquely contribute to death anxiety beyond psychological distress among hemodialysis patients, particularly in non-Western and culturally religious contexts.

### Aim of the Study

The present study aims to (1) assess levels of spiritual needs, psychological distress, and death anxiety among individuals receiving hemodialysis in Eastern Turkey; (2) examine the associations among these variables; and (3) determine whether spiritual needs independently predict death anxiety beyond the effects of psychological distress in this population.

## Methods

### Study Design

A cross-sectional and correlational research design was used in this study. Throughout the study, the authors followed the Strengthening the Reporting of Observational Studies in Epidemiology (STROBE) guidelines and prepared the report accordingly (Von Elm et al., 2007). This study was conducted between October and November 2024 at a hemodialysis center in Eastern Turkey. Data were collected from 131 patients receiving hemodialysis treatment via self-report questionnaires administered during routine dialysis sessions. Participant eligibility was assessed in collaboration with dialysis nursing staff. Participation was voluntary, written informed consent was obtained from all participants, and patients who withdrew consent or provided incomplete data were excluded from the final analyses.

### Population and Sample

The study population consisted of patients receiving hemodialysis at a dialysis center in Eastern Turkey between October and November 2024. At the time of data collection, 169 patients were registered at the dialysis unit. The sample size was determined via a finite population formula with a 95% confidence level and a 5% margin of error, assuming a response distribution of 50% to maximize variance. On the basis of this calculation, the minimum required sample size was 118 participants. A total of 131 patients meeting the eligibility criteria were recruited, providing sufficient statistical power. The inclusion criteria were as follows: aged 18 years or older; diagnosed with chronic renal disease; received hemodialysis for at least three months; able to read, write, and understand Turkish; and were physically stable (no severe sensory impairments or lethargy), emotionally stable at the time of data collection. Patients with a documented diagnosis of psychiatric disorders, cognitive impairment, or intellectual disability were excluded on the basis of self-reports and medical records. Participant eligibility was assessed in collaboration with the dialysis nursing staff. Individuals who withdrew consent or, submitted incomplete questionnaires were excluded from the final analysis.

### Data Collection Tools

The data were collected as follows:

1. Sociodemographic and Medical Information Form
2. Patient Spiritual Needs Assessment Scale (PSNAS)
3. The Depression Anxiety Stress Scale–21 (DASS-21)
4. The Templer Death Anxiety Scale (T-DAS)

No scale development, translation, or psychometric validation procedures were conducted as part of this study.

### Sociodemographic and Medical Information Form

This researcher-developed form included 10 items assessing sociodemographic characteristics (age, sex, marital status, education, employment) and clinical information (disease duration, hemodialysis duration, comorbidities, and relevant medical history).

### Patient Spiritual Needs Assessment Scale (PSNAS)

The Patient Spiritual Needs Assessment Scale was used to assess participants’ spiritual needs. To enhance transparency and allow readers to evaluate item-level content, a full list of PSNAS items in English is provided in Appendix 1. The Turkish adaptation and psychometric validation of the PSNAS were conducted by Dedeli et al. (2015).

In response to recent concerns regarding measurement contamination in spirituality and mental health research (Koenig & Carey, 2024), only PSNAS subscales that do not conceptually overlap with psychological well-being, emotional functioning, or mental health constructs were retained for analysis. Specifically, three subscales were included:

- The Divine—assessing needs related to connection with God or transcendent beliefs
- Appreciation of beauty—assessing sensitivity to beauty, art, and sacred experiences
- Death and Resolution—assessing spiritual concerns related to death, meaning, and existential closure

Subscales measuring meaning and purpose, love and belonging, and positivity/gratitude/hope/peace were excluded because of their conceptual overlap with psychological well-being and emotional adjustment to minimize contamination with depression, anxiety, and stress measures.

The items were rated on a 4-point Likert scale ranging from 1 (“never”) to 4 (“very often”), with higher scores indicating greater levels of spiritual needs. A composite uncontaminated spiritual needs score (PSNAS-Clean) was calculated by summing the retained subscale scores. In the present study, the internal consistency of the PSNAS-Clean composite was excellent (Cronbach’s α = 0.85), indicating strong reliability.

This analytic approach provides a more conservative and methodologically rigorous assessment of spiritual needs by isolating spiritual domains that are not confounded by emotional well-being or mental health symptom content.

### Depression Anxiety Stress Scale–21 (DASS-21)

Psychological distress was assessed via the Depression Anxiety Stress Scale–21 (DASS-21), a widely used self-report instrument that measures symptoms of depression, anxiety, and stress. The scale includes 21 items, with 7 items per subscale, rated on a 4-point Likert scale from 0 (“Did not apply to me at all”) to 3 (“Applied to me very much or most of the time”)**.** The Turkish validity and reliability study was conducted by Sarıçam (2018)**.** Subscale scores were calculated according to standardized scoring procedures. In the present study, the overall internal consistency of the DASS-21 was high (Cronbach’s α = 0.91).

### Templer Death Anxiety Scale (T-DAS)

Death anxiety was measured via the Templer Death Anxiety Scale (T-DAS), which was originally developed by Templer (1970) and adapted into Turkish by Akça and Köse (2008). The scale consists of 15 dichotomous (true/false) items, with total scores ranging from 0 to 15, where higher scores indicate greater death anxiety. In the current sample, internal consistency was strong (Cronbach’s α = 0.87).

### Implementation and Ethical Considerations

Ethical approval was obtained from the Ethics Committee of XXX (Approval No: 2024/10–14; Date: 20/09/2024). Institutional permission was granted by the dialysis center administration. All participants provided written and verbal informed consent prior to participation.

Data collection took place during routine hemodialysis sessions in a quiet and private area of the treatment unit. The participants were informed that their responses would remain confidential and would be used solely for research purposes. Completing the questionnaires required approximately 20–25 min**.**

### Statistical Analysis

The data were analyzed via IBM SPSS Statistics version 30.0. Normality was assessed via the Kolmogorov–Smirnov test. Although distributions deviated from normality, parametric tests were employed owing to the sample size and robustness of correlation and regression analyses.

Descriptive statistics (means, standard deviations, frequencies, and percentages) were calculated for sociodemographic variables and scale scores. Pearson correlation analysis was used to examine associations among spiritual needs, psychological distress (stress, anxiety, depression), and death anxiety.

To test the primary study hypothesis, a hierarchical multiple regression analysis was conducted. In Step 1, depression, anxiety, and stress were entered as covariates. In Step 2, spiritual needs (PSNAS) were added to determine whether spiritual needs predict death anxiety beyond psychological distress. Multicollinearity diagnostics indicated acceptable variance inflation factor (VIF) values. Statistical significance was set at *p* < .05**.**

## Results

The personal characteristics of the participants are presented in Table 1. More than half of the participants (56.5%) were aged over 50 years. The majority were male (61.1%) and married (63.4%). Over half (53.4%) reported having a poor level of income. Approximately 29% of the participants had been diagnosed with chronic kidney disease for 1–5 years, whereas 30.5% had been receiving hemodialysis treatment for the same duration. In addition, 60.3% of the participants reported having at least one other chronic disease.

**Table 1:** Participant characteristics (N = 131)

		N	%
Age	18–30	15	11.5
	30–50	42	32.1
	Above 50	74	56.5
Gender	Male	80	61.1
	Female	51	38.9
Marital Status	Single	30	22.9
	Widow	18	13.7
	Married	83	63.4
Economic Status	Income less than expenses	70	53.4
	Income more than expenses	15	11.5
	Income equal to expenses	46	35.1
Education Status	Literate	45	34.4
	Primary School	34	26.0
	Secondary School	20	15.3
	High school	25	19.1
	Bachelor’s degree	7	5.3
Length of having chronic renal failure (years)	< 1	9	6.9
	1–5	38	29.0
	5–7	20	15.3
	7–10	29	22.1
	Above 10	35	26.7
Length of getting dialysis treatment	< 1	13	9.9
	1–5	40	30.5
	5–7	25	19.1
	7–10	29	22.1
	Above 10	24	18.3
Having a kidney transplant	Yes	13	9.9
	No	118	90.1
Having physical chronic illness	Yes	79	60.3
	No	52	39.7
Chronic illness	Diabetes	25	20.3
	Hypertension	36	27.6
	Heart failure	4	3.1
	COPD	4	3.1
	Epilepsy	3	2.3
	Other (Vision Loss, Hep C, Pancreas, Urticaria, Rheumatism, Cancer Prostate-Intestinal etc.)	8	5.6

The descriptive statistics for the participants’ scale scores are presented in Table 2. Descriptive values are also reported for PSNAS subscales that were excluded from inferential analyses owing to conceptual overlap with mental health constructs. These subscale scores are provided for transparency but were not interpreted analytically.

**Table 2:** Descriptive statistics for death anxiety, spiritual needs subscales, and psychological distress (N = 131)

Variable	Minimum	Maximum	Mean	SD
Death anxiety (T-DAS)	0.00	12.00	8.80	1.89
*Spiritual needs subscales (PSNAS)*				
Divine †	0.00	12.00	7.98	3.49
Appreciation of beauty †	1.00	9.00	6.04	2.19
Death and resolution †	0.00	12.00	7.99	3.02
Meaning and purpose ‡	0.00	9.00	5.59	2.56
Love and belonging ‡	2.00	15.00	11.51	3.31
Positivity/gratitude/hope/peace ‡	1.00	12.00	9.06	2.77
PSNAS total score	15.00	69.00	48.17	13.58
PSNAS-Clean composite †	4.00	33.00	22.01	6.71
*Psychological distress (DASS-21)*				
Stress	1.00	18.00	8.45	3.59
Anxiety	0.00	17.00	6.72	3.65
Depression	0.00	20.00	6.95	4.41

^†^ Subscales included in inferential analyses (uncontaminated spiritual needs)

^‡^ Subscales reported for descriptive purposes only owing to conceptual overlap with mental health constructs

PSNAS-Clean = composite score derived from the Divine, Appreciation of Beauty, and Death and Resolution subscales

SD = standard deviation

With respect to spiritual needs, the mean score on the uncontaminated PSNAS composite was 22.01 (SD = 6.71; range = 4–33). Among the retained PSNAS subscales, the mean score for the divine domain was 7.98 (SD = 3.49), the mean score for death and resolution was 7.99 (SD = 3.02), and the mean score for appreciation of beauty was 6.04 (SD = 2.19).

Subscales excluded from primary analyses due to potential conceptual overlap with mental health constructs (meaning and purpose, love and belonging, and positivity/gratitude/hope/pace) yielded mean scores of 5.59, 11.51, and 9.06, respectively, but were not retained in inferential analyses to minimize measurement contamination.

The mean score on the Templer Death Anxiety Scale was 8.80 (SD = 1.89; range = 0–12).

The mean stress score was 8.45 (SD = 3.59; range = 1–18), the mean anxiety score was 6.72 (SD = 3.65; range = 0–17), and the mean depression score was 6.95 (SD = 4.41; range = 0–20).

The relationships between spiritual needs, spiritual well-being, depression, stress and anxiety and death anxiety are shown in Table 3.

**Table 3:** Correlations between variables

Variable	1	2	3	4	5	6	7	8
1. Divine	–							
2. Appreciation of beauty	0.21*	–						
3. Death and resolution	0.49**	0.41**	–					
4. PSNAS-Clean (core spiritual needs)	0.81**	0.62**	0.84**	–				
5. Anxiety	0.07	− 0.03	0.24**	0.14	–			
6. Stress	− 0.09	0.14	0.26**	0.12	0.51**	–		
7. Depression	− 0.16	− 0.08	0.10	− 0.06	0.59**	0.57**	–	
8. Death anxiety (T-DAS)	0.13	0.28**	0.39**	0.33**	0.20*	0.15	0.08	–

^*^p < 0.05; **p < 0.001, Pearson correlation was used

Pearson correlation analyses indicated that uncontaminated spiritual needs were positively associated with death anxiety (r = 0.33, *p* < .001). Among the PSNAS subscales, the death and resolution domain demonstrated the strongest correlation with death anxiety (r = 0.39, *p* < .001), followed by appreciation of beauty (r = 0.28, *p* = 0.001). The Divine subscale was not significantly correlated with death anxiety (r = 0.13, *p* = 0.149).

The PSNAS-Clean score was not significantly correlated with depression, anxiety, or stress, indicating that there was no statistically significant association between uncontaminated spiritual needs and psychological distress in this sample.

Death anxiety showed a small positive correlation with anxiety (r = 0.20, *p* = 0.025), whereas its correlations with stress and depression were not statistically significant.

A hierarchical multiple regression analysis was conducted to examine whether uncontaminated spiritual needs predicted death anxiety after controlling for psychological distress (see Table 4).

**Table 4:** Hierarchical regression predicting death anxiety (N = 131)

Predictor	B	SE	β	t	*p*	ΔR^2^
*Step 1*						0.047
Stress	0.052	0.058	0.10	00.90	0.371	
Anxiety	0.106	0.058	0.21	10.84	0.069	
Depression	− 0.042	0.050	− 0.10	− 00.84	0.401	
*Step 2*						0.088*
Stress	0.024	0.056	0.05	00.42	0.672	
Anxiety	0.071	0.056	0.14	10.26	0.209	
Depression	− 0.004	0.049	− 0.01	− 00.07	0.943	
PSNAS-Clean	0.086	0.024	0.31	30.57	< 0.001	

Dependent Variable = Death anxiety (T-DAS). ΔR^2^ = change in explained variance at Step 2

^*^p < 0.05

In Step 1, anxiety, stress, and depression did not significantly predict death anxiety, R^2^ = 0.047, F = 2.08, *p* = 0.107.

In Step 2, uncontaminated spiritual needs (PSNAS-Clean) were added to the model, resulting in a significant increase in explained variance, ΔR^2^ = 0.088, F-change = 12.75, *p* < 0.001. In the final model (R^2^ = 0.134), spiritual needs were a significant independent predictor of death anxiety (B = 0.086, SE = 0.024, β = 0.31, *p* < 0.001), whereas anxiety, stress, and depression were not statistically significant predictors.

These results indicate that uncontaminated spiritual needs account for additional variance in death anxiety beyond psychological distress in this sample.

## Discussion

This study examined spiritual needs, psychological distress (depression, anxiety, stress), and death anxiety among patients undergoing hemodialysis in Eastern Turkey, with a particular emphasis on uncontaminated spiritual needs derived from the Patient Spiritual Needs Assessment Scale. By focusing on spiritual concerns that are conceptually distinct from mental health constructs, the findings contribute to ongoing efforts to improve the methodological rigor of spirituality and health research.

The participants reported substantial spiritual needs, particularly in domains related to relational connectedness, hope and positivity, and concerns surrounding death and existential resolution. These patterns are consistent with prior research in populations living with chronic or life-threatening conditions, suggesting that meaning making, belonging, and preparation for mortality constitute central spiritual themes in medically vulnerable groups (Dedeli et al., 2015; Faurizi et al., 2017). Ongoing illness burden, uncertainty regarding prognosis, and repeated exposure to medical stressors may intensify the need for emotional closeness, existential reassurance, and spiritual coping resources, underscoring the relevance of spiritual care within clinical settings (Musa et al., 2018; Hassani et al., 2022).

Psychological distress was also evident in this sample, with participants reporting mild to moderate levels of depression, anxiety, and stress. This finding aligns with the literature documenting elevated mental health burdens among individuals receiving hemodialysis (Dehghan et al., 2021; Musa et al., 2018). Chronic disease management, lifestyle limitations, treatment dependence, and uncertainty about future health outcomes may contribute to sustained emotional strain, reinforcing the need for routine mental health screening and psychosocial support in dialysis care.

Death anxiety has emerged as a prominent concern. The mean scores exceeded commonly used clinical thresholds, which is consistent with prior findings among hemodialysis patients in Iran and Palestine (Ghiasi et al., 2021; Ibrahim et al., 2023). Living with a life-limiting illness, recurrent exposure to invasive procedures, and heightened awareness of mortality may increase existential vulnerability and fear of death. Elevated death anxiety may, in turn, have implications for emotional well-being, treatment engagement, and quality of life.

A notable methodological contribution of this study is the use of a contamination-adjusted measure of spiritual needs. Unlike spirituality scales, which embed emotional well-being or mental health content, the PSNAS-Clean composite focuses on core existential, relational, and transcendent concerns. Correlational analyses indicated that uncontaminated spiritual needs were not significantly associated with depression, anxiety, or stress, supporting the conceptual distinctiveness of the construct. This finding aligns with recent critiques emphasizing the risk of construct contamination in spirituality research and the importance of analytically separating spiritual and psychological domains (Koenig, 2012; Dedeli et al., 2015).

Hierarchical regression analysis further demonstrated that uncontaminated spiritual needs significantly predicted death anxiety even after controlling for depression, anxiety, and stress. Psychological distress did not independently explain death anxiety, whereas spiritual needs accounted for additional variance. This pattern suggests that concerns related to meaning, mortality, belonging, and existential resolution may contribute to fear of death in ways that are not reducible to general emotional distress. Rather than representing a proxy for psychopathology, spiritual needs appear to function as a distinct experiential domain relevant to end-of-life concerns.

Among the spiritual dimensions, the death and resolution subscale had the strongest association with death anxiety. Patients who reported greater struggle with existential closure or unresolved concerns related to death may experience heightened mortality-related fear. This observation is consistent with prior research indicating that existential uncertainty and unresolved spiritual concerns can intensify death-related distress (Feng et al., 2021; Ghiasi et al., 2021). Interventions that provide opportunities for meaning-centered reflection, spiritual counseling, or supportive discussion of death-related concerns may therefore hold promise for alleviating death anxiety in this population.

Taken together, the findings highlight spiritual needs as an important dimension of holistic care that is conceptually distinct from psychological distress yet meaningfully related to existential fear. The incorporation of spiritually sensitive approaches—such as facilitating meaning-making, relational connections, and open dialog about mortality—may support emotional adaptation and well-being among patients undergoing hemodialysis.

## Limitations

This study has several limitations that should be considered when interpreting the findings. First, the cross-sectional design precludes causal inference, and longitudinal research is needed to clarify the temporal relationships among spiritual needs, psychological distress, and death anxiety. Second, the study was conducted at a single dialysis center with a modest sample size, which may limit generalizability. Third, the data were based on self-report measures, which are susceptible to response bias and social desirability effects. Finally, although the study employed a contamination-adjusted spirituality measure, future research may benefit from combining quantitative assessments with qualitative approaches to deepen the understanding of existential and spiritual experiences among hemodialysis patients.

## Conclusion

This study demonstrated that hemodialysis patients in Eastern Turkey experience high levels of death anxiety and substantial spiritual needs, particularly in domains related to meaning, connection, and death-related concerns. Crucially, spiritual needs predict death anxiety independently of depression, anxiety, and stress**,** suggesting that existential concerns represent a unique and clinically relevant dimension of patient experience.

These findings underscore the importance of incorporating spiritual and existential assessment into nephrology and dialysis care alongside routine psychological screening. Healthcare professionals may support patients by facilitating conversations about meaning, mortality, and connection, as well as by offering them access to culturally appropriate spiritual resources. Addressing spiritual needs in a sensitive and individualized manner has the potential to reduce death-related distress, strengthen coping, and improve overall quality of life among hemodialysis patients.

## Appendix 1

English Items of the Patient Spiritual Needs Assessment Scale (PSNAS), Organized by Subscale.

Items excluded from PSNAS-Clean are marked with an asterisk (*).

Response format: 1 = Never, 2 = Rarely, 3 = Fairly often, 4 = Very often.

The Divine (Retained in PSNAS-Clean).

**Table:**

Item
To have a closer relationship with God
To pray
To feel God’s presence
To trust in God
To talk with someone about God

Appreciation of Beauty (Retained in PSNAS-Clean)

**Table:**

Item
To appreciate beauty in nature
To enjoy art, music, or literature
To feel inspired by beauty
To experience sacredness in ordinary things

Death and Resolution (Retained in PSNAS-Clean)

**Table:**

Item
To address issues before death and dying
To address concerns about life after death
To review your life
To forgive yourself and others

Meaning and Purpose (Excluded from PSNAS-Clean)

**Table:**

Item
*To find meaning in life
*To understand the purpose of suffering
*To feel that life has meaning
*To understand why things, happen

Love and Belonging (Excluded from PSNAS-Clean)

**Table:**

Item
*For companionship
*For compassion and kindness
*To feel loved
*To feel a sense of connection with others

Positivity/Gratitude/Hope/Peace (Excluded from PSNAS-Clean)

**Table:**

Item
*To be thankful or grateful
*To feel hopeful
*To keep a positive outlook
*To feel a sense of peace and contentment
